# Predicting the Response of RC Beam from a Drop-Weight Using Gene Expression Programming

**DOI:** 10.3390/ma15196910

**Published:** 2022-10-05

**Authors:** Moiz Tariq, Azam Khan, Asad Ullah

**Affiliations:** NUST Institute of Civil Engineering (NICE), School of Civil and Environmental Engineering, National University of Science and Technology (NUST), Sector H-12, Islamabad 44000, Pakistan

**Keywords:** gene expression programming (GEP), reinforced concrete beam, impact loading, statistical analysis, numerical simulation

## Abstract

For structures and load-bearing beams under extreme impact loading, the prediction of the transmitted peak impact force is the most challenging task. Available numerical and soft computing-based methods for finding peak impact force are not very accurate. Therefore, a simple and user-friendly predictive model is constructed from a database containing 126 impact force experiments of the simply supported RC beams. The proposed model is developed using gene expression programming (GEP) that includes the effect of the impact velocity and the impactor weight. Also identified are other influencing factors that have been overlooked in the existing soft computing models, such as concrete compressive strength, the shear span to depth ratio, and the tensile reinforcement quantity and strength. This allows the proposed model to overcome several inconsistencies and difficulties residing in the existing models. A statistical study has been conducted to examine the adequacy of the proposed model compared to existing models. Additionally, a numerical confirmation of the empirical model of the peak impact force is obtained by reference to 3D finite element simulation in ABAQUS. Finally, the proposed model is employed to predict the dynamic shear force and bending moment diagrams, thus rendering it ideal for practical application.

## 1. Introduction

The complex behavior of reinforced concrete (RC) beams under impact loading has received significant attention over the past few decades [1,2,3,4]. Various response parameters are responsible for characterizing the dynamic response of impacted RC beams [5,6,7,8,9]. One of the most important parameters is the peak impact force, whose correct prediction is essential to designing optimized structural elements that can mitigate the hazardous effects [10,11,12,13,14]. A laboratory test method to measure the peak impact force is the drop weight impact test, in which the contact force between the impactor and the beam is determined by a load cell [15,16,17]. Once the peak impact force is known, the bending moment and the shear force can be easily determined [18]. So the knowledge of peak impact force determines the whole dynamic behavior of impacted RC beams. Recognizing the significance of the peak impact force, several analytical and computation models have been developed in the past to reliably predict this force. However, most of the available techniques require expertise and are computationally expensive [19,20]. Evolutionary algorithms have proven to be a pertinent technique for developing efficient predictive models that are also user-friendly.

Developing an efficient and accurate predictive model requires an in-depth understanding of key influencing parameters. To establish these parameters concerning the peak impact force, it is increasingly important to understand the overall behavior of impacted RC beams. When impact load is applied to these beams, they undergo two response phases, namely a local phase and a global phase. The local response is identified by a localized shear plug resulting in local deformation at the point of impact, whereas the global phase is identified by a flexure plastic hinge at the point of impact resulting in global deflection of the beam [3,10,20,21,22,23,24,25,26,27,28,29,30,31,32,33]. From this standpoint, two fundamental response parameters sufficient for adequate prediction of the dynamic response of RC beams can be emphasized: (1) the impact force submitted to the beam and (2) the maximum deflection of the beam. The impact force and the maximum deflection are normally predicted by using various numerical, analytical, or empirical methods. One of the most simple and accurate methods of predicting the response of structures under extreme dynamic loading is based on the rigid-plastic theory [20].

Several experimental investigations have been carried out on RC elements [4,13,15,21,22,23,24] under impact loading. These results are also verified by employing commercial software packages, such as ABAQUS and LS-DYNA [10,18,25,26,27,28,29], which can be computationally expensive and time-consuming [1]. Computational processes based on evolutionary algorithms have grown out of a recognition of these inadequacies of traditional design techniques. Genetic algorithms, evolutionary computing, and neural networks have made significant inroads into the analysis and design of structural systems. These techniques can be successfully employed to develop equations that can forecast the important response parameters, such as peak impact force.

Pham et al. [3] developed a neural network-based impact force model for reinforced concrete beams. Similarly, Zhao et al. [7] proposed an analytical model based on the contact laws and the conservation of energy to predict the peak impact response of RC beam under impact loading. Additionally, Tachibana et al. [23] proposed an equation to predict the maximum displacement of RC beam and compared the results with experiments and FEM simulation. In addition, Kishi and Mikami [2] proposed empirical relations for flexural load-carrying capacity and the maximum displacement for the reinforced concrete beam. Ando et al. [30] and Kishi et al. [31] carried out an experimental investigation and concomitant empirical analysis of RC beams subject to input impact energy.

Nevertheless, all foregoing soft computing models [3] have been developed using limited influencing factors, and the available numerical models [21,22,23,24] pose excessive computational complexity, which prevents the common application of these models. Similarly, the drawback of the available analytical models [7] is that they are too simplistic, mainly based on single degree of freedom dynamic systems. These considerations require the development of a feasible and efficient model that can predict the response in the spirit of the available experimental data. The current study aims to create a robust model using gene expression programming for predicting the peak impact force on the RC beam from drop-weight. The accuracy of the model is assessed using experimental observations contained in an extensive database containing 126 impact force experiments. This study proceeds by providing a parametric equation of the peak impact force whose results are compared with the existing models in the literature. Additionally, a numerical simulation is presented for two of the experimental tests on the RC beam and its predictions compared with the proposed empirical model of the impact force. This force can also be employed to predict the shear force and the bending moment diagrams for the RC beams under impact loading. For this reason, it is asserted that the drawbacks in the existing models are overcome by developing the current user-friendly GEP model that incorporates additional influencing parameters, such as the shear span to depth ratio, the tensile reinforcement, and the shear reinforcement.

## 2. Research Significance

One of the most important response parameters is the peak impact force, which is generated within a beam as a result of the applied impact and governs the overall dynamic response of the impacted beam. The peak impact force from a laboratory experiments is calculated using an expensive load cell and sensor assembly. Once the peak impact force is obtained, the dynamic shear force and the bending moment diagrams can be easily determined, thereby offering all the necessary information to design the impacted beam. To avoid the expensive impact force assembly, very limited models [4,13,15,21,22,23,24] are available to predict the peak impact force but with insufficient accuracy. The current study aims at identifying some key factors that the available models have overlooked, such as concrete compressive strength, the shear span to depth ratio, and the tensile reinforcement quantity and strength. Hence, a robust regression-based model [3] is built that incorporates all these key parameters, resulting in an accurate prediction of peak impact force, which compensates for the inconsistencies of the available models. The predictive ability of the proposed gene expression programming-based model is ensured by comparing the results with the existing soft computing models and single degree of freedom analytical models [7].

## 3. Assumptions

The proposed method is intended for predicting the peak impact force transferred to a simply supported beam subjected to midspan drop weight impact. The following assumptions are made during the development of the new model:

1. The shape of the impactor is considered not to influence the impact behavior of RC beams;
2. The impact event is so fast that the damping of the impacted component can be neglected;
3. The bearing plates are normally provided to distribute the impact load and avoid localized failure. This influence of the bearing plate is ignored;
4. The impact force is applied perpendicular to the longitudinal axis of the beam;
5. No provision is made in the proposed model for incorporating special anchored reinforcement.

## 4. Discussion of Influencing Parameters

In setting up an empirical model for the impact force on RC beams, the knowledge of various influencing parameters is essential. Therefore, various important factors are identified [8,11,32,33,34], such as the concrete compressive strength, the longitudinal tensile reinforcement, the yield strength of longitudinal reinforcement, the vertical shear reinforcement, the mass, the velocity of the drop-weight, and different geometric properties of the RC beam. The significance of these variables has been demonstrated by extensive experimental studies by Hughes and Mahmoud [35], Louw et al. [36], May et al. [37], Zhan et al. [38], and Goldston et al. [13]. From these precedents, Zhan et al. [38], May et al. [37], and Louw et al. [36] pointed out that the concrete compressive strength shows a positive correlation with the impact force from a drop-weight. A similar increasing trend has been observed by Adhikary et al. [32] through numerical simulation. This trend follows intuition since as the compressive strength is increased, the beam capacity and the stiffness are increased, thereby resulting in an increased peak impact force because stiffer elements attract more force. In the same context, increasing the width or depth of the beam will attract more impact force than increasing the length of the beam.

Longitudinal tensile reinforcement is another important parameter affecting the peak impact force on the RC beam. In this instance, Goldston et al. [13], Hughes and Mahmoud [35], Adhikary et al. [11], and Zhan et al. [38] observed the influence of the longitudinal tensile reinforcement on the impact force because as the tensile reinforcement increases, both the ultimate bending moment capacity as well as the ultimate load-carrying capacity increases. Zhan et al. [38] and Fujikake et al. [4] also observed that the stiffness of the RC beam increases with the increase in the longitudinal tensile reinforcement, which tends to rise the peak impact force. The yield strength of the longitudinal reinforcement also contributes to the impact force as it increases the brittleness of the RC beam. Similar behavior has been produced by Adhikary et al. [32] with the help of a numerical study. Adhikary et al. [11] and Bhatti et al. [8] have shown the increasing trend of the peak impact force with the increasing vertical shear reinforcement. This trend is due to the transverse reinforcement providing additional confinement to the core concrete, thereby supplementing lateral restraint capacity against the buckling of the longitudinal reinforcements.

Input kinetic energy ($K.E\;=\;\frac{1}{2}{MV}^{2}$) has a vital role in the impact analysis of the RC beam under drop-weight. By increasing the input kinetic energy, the increase in the peak impact load has been reported by various researchers [8,33,34].

## 5. Experimental Database

The peak impact force prediction model has been developed using an extensive database of 126 experimental tests from the previous studies [2,4,8,23,24,38,39,40,41,42], summarized in Table A1 and Table A2. This database comprises RC beams of rectangular cross-sections subjected to impact, where the impactor’s contact surface is either flat or spherical. For GEP analysis, only a randomly selected portion of 84 experiments is used to develop the model and the remaining 42 experiments are used to validate the model.

### Distribution of Key Influence Parameters

The dataset comprises various influencing parameters that are essential for developing a robust predictive model. The determination of these parameters requires an in-depth review of experimental investigations. The parameters influencing the peak impact force and their validity ranges are derived from the literature [4,13,15,21,22,23,24]. These key parameters are the impact velocity, the impact mass, the geometric size of the beam, the concrete compressive strength, the longitudinal reinforcement, and the shear reinforcement. These parameters, along with their respective ranges within the dataset, are listed in Table 1.

## 6. Previous Models to Evaluate the Peak Impact Force on RC Beam

The reliability and validity of any model can be demonstrated by comparing it with the existing analytical and numerical models. Hence, the models proposed by Zhao et al. [7] and Pham and Hao [3] are selected for this purpose.

### 6.1. Zhao et al. Model

Zhao et al. [7] proposed a peak impact force model using the contact law and the conservation of energy. In this model, the energy-balance equation for predicting the peak impact force for both spherical and flat nose drop-weight impactors can be given by the following equations, respectively:

(1) $$F_{P}={\left[\frac{9MV^{2}}{8\left(\frac{M}{m+1}\right)\;}{\left(\frac{\pi ER}{d}\right)}^{\frac{1}{2}}\right]}^{\frac{2}{3}}$$

(2) $$F_{P}={\left(\frac{3MV^{2}}{4\left(\frac{M}{m+1}\right)}\times \frac{EA}{d}\right)}^{\frac{1}{2}}$$

where $F_{P}$ is the impact force, M and V are the mass and velocity of the impactor, m is the effective mass of the beam (calculated using the approximate design method proposed by Biggs), E is the elastic modulus of concrete (where $E=5000\times \surd f_{c}^{\prime }$), d is the depth of the beam, and R and A are the respective radius and area of the impactor.

### 6.2. Pham and Hao Model

Pham and Hao [3] introduced an artificial neural network (ANN)-based empirical model. The simplified form of the model is given below;

(3) P=W×X+e

where W is a proportional matrix, X is the input matrix, and e is scalar, which is calculated as follows:

(4) $$e=LW\times b_{1}+b_{2}$$

(5) $$W=LW\times IW=\left[w_{1}\;w_{2}\;w_{3}\;w_{4}\;w_{5}\;w_{6}\right]$$

(6) $$X={\left[x_{1}\;x_{2}\;x_{3}\;x_{4}\;x_{5}\;x_{6}\right]}^{T}={\left[f_{c}^{\prime }\frac{EI}{L_{n}}\;m\;m_{2}\;M\;V\right]}^{T}$$

where IW is the input weight matrix, $b_{1}$ is the bias matrix of layer 1, LW is the layer weight matrix, $b_{2}$ is the bias matrix of layer 2, $f_{c}^{\prime }$ is the concrete compressive strength, $\frac{EI}{L_{n}}$ is the flexural stiffness parameter with *EI* as flexural rigidity and $L_{n}$ as the net span, m is the beam weight, $m_{2}$ is the weight of the overhanging ends of beam, M is the impactor mass, and V is the impactor velocity. Additionally, *x* means the cross product.

The above model can be simplified in the following form:

(7) $$P=\overset{6}{\underset{i=1}{\sum }}k_{i}x_{i}+c$$

where $k_{i}$ are proportional factors and c is a constant. In this model, it is derived that *c* = 21,119 and

(8) k=[7.63  −0.02   1.93  −3.17   1.34   186.09]

## 7. GEP Algorithm

Gene expression programming (GEP) is a type of genetic algorithm (GA) that generates mathematical models from the input data and processes them in a domain-independent manner. In terms of chromosome representation, GEP differs from the genetic algorithms GAs and the genetic programming GP. In GAs, chromosomes are linear strings of fixed length, whereas in GPs, they are nonlinear entities of varying sizes and shapes [43,44,45]. GEP, on the other hand, encompasses both a fixed-length linear string and a ramified structure of various sizes and shapes.

As with other evolutionary algorithms, several trials were carried out to execute the evolutionary process by iteratively changing the number of chromosomes, the number of genes, the size of the head, and the linking functions. Thus, GEP optimizes solutions by selecting the best candidates from the provided initial population based on their fitness. Notably, increasing the number of genes and chromosomes can result in a complicated function but the function can precisely fit the results. There is a trade-off between achieving a simplified mathematical model by controlling the number of genes and chromosomes and achieving the desired level of accuracy [43,44,45,46,47].

Convergence to the global optimal solution is a critical step in the GEP algorithm. The algorithm may fail to select an optimal solution from among several competing candidate solutions at times. In this state, the algorithm can lead to an indefinite sequence of steps, which can result in either a non-terminating program or an illogical expression. This problem can be solved by adjusting the linking function or changing the number of genes and chromosomes [48].

Over the last decade, the advantages of the GEP have attracted widespread application in the field of structural engineering. Several authors [3] have used the GEP to develop models for estimating the capacity of various structural components elegantly. In the present work, the GEP was used successfully to predict the behavior of an RC beam from a drop-weight.

**Figure 1:**
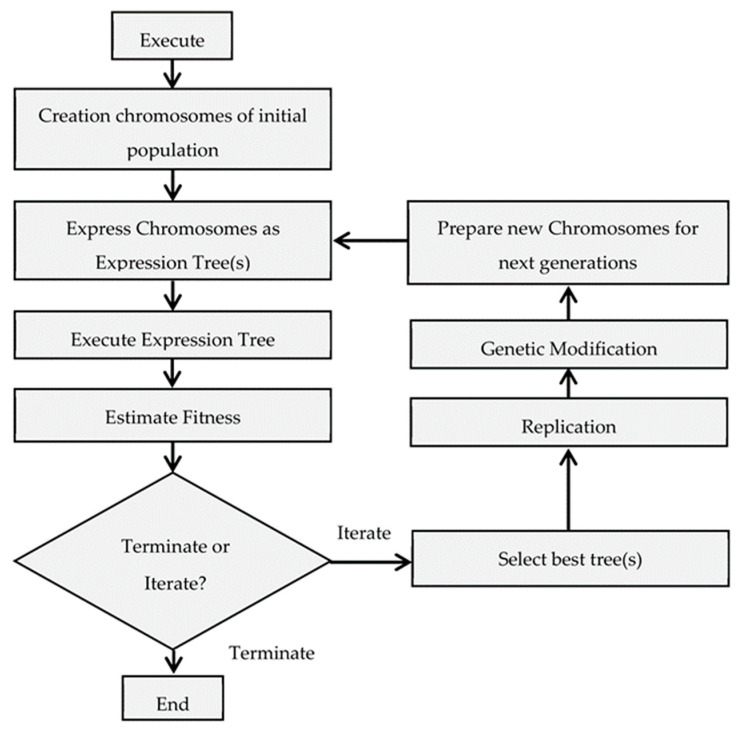
Flow chart for GEP model construct.

Figure 1 depicts the various stages of GEP optimization. The optimization procedure begins with the selection of control parameters, such as the function set, the terminal set, the fitness function, the control parameters, and the stop condition. The fitness function is specified before the execution of the evolutionary algorithm, which results in the creation of a random string of initial population, also known as ‘chromosomes’ in genetic programming parlance. These strings are translated into an expression tree, the results of which are compared to each chromosome’s fitness score. If the fitness criterion is not met, a roulette-wheel sampling method is used to select some chromosomes, which are then mutated to produce new generations. In contrast, if the variables are optimally tuned to the fitness function, the chromosomes are optimized [49].

## 8. Proposed GEP Model for Estimating Peak Impact Force

In this section, a GEP model is proposed for the peak impact force on the RC beam. The GEP models generated from the previously mentioned dataset can be represented by the following equation extracted from genetic sub-elemental tail (Sub ET):

(9) $$F_{p}\left(kN\right)=G1+G2+G3\;$$

(10) $$G1={\left(\frac{1}{3.07+2V}\right)}^{\frac{5}{2}}+\left(\frac{V}{L_{n}+\frac{A_{s}f_{y}}{f_{c}^{\prime }}+s-2390.26}\right)$$

(11) $$G2=\left[\left\{\frac{f_{c}^{\prime }}{\frac{a}{d}-2.93}+\left(\ln\left(V\right)\times \left(b-\rho_{v}f_{y}+40.27\right)\right)\right\}-\left(f_{c}^{\prime }\times \sqrt{V}\right)\right]$$

(12) $$G3=\left\{\left(\left(6.83+f_{c}^{\prime }\right)\times V\right)+b+3h-\frac{a}{d}-\frac{M}{V}-2s-d-404.96\right\}$$

where b, h, and d are the respective width, height, and effective depth of the beam; V and M are the velocity and the mass of drop-weight, respectively; $L_{n}$ and $\frac{a}{d}$ represent the respective net span and shear span to depth ratio of the beam; $A_{s}f_{y}$ is the product of area and yield strength of longitudinal reinforcement; ln is the natural log; $f_{c}^{\prime }$ is the concrete compressive strength; $\rho_{v}f_{y}$ is the product of reinforcement ratio and yield strength of transverse reinforcement; and s is the mid-span deflection.

**Figure 2:**
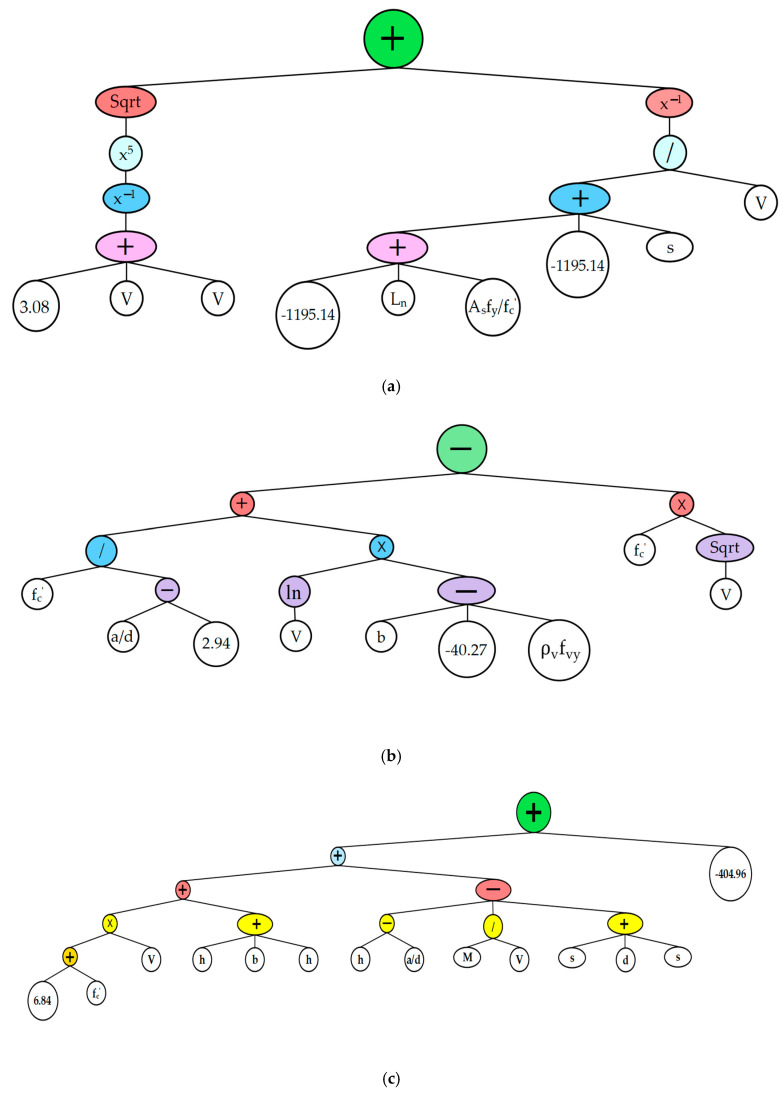
Gene expression for the peak impact force. (**a**) Sub ET-1 (**b**) Sub ET-2 (**c**) Sub ET-3.

The expression tree of the estimation model is also given in Figure 2, along with the model construction parameters shown in Table 2.

## 9. Accuracy of the Proposed Model

The accuracy of any empirical model is inextricably linked to the training and validation of these models using the available dataset [45]. In the case of the current model, a random sample of 60% from the designated RC beam experiments is used as training data (for model creation), while a random sample of 40% from each category is used for validation. After the model development, several statistical performance checks, such as the performance factor (PF), coefficient of variation (*CoV*), average absolute error (*AAE*), and coefficient of determination are used to gain quantitative insight into the model. The coefficient of variation ( *CoV*) measuring dispersion around data mean is the precision testing measure that can be presented by

(13) $$CoV\;\left(\%\right)=\left\{\frac{Standard\;Deviation\;\left(\sigma \right)}{Mean\;\left(\mu \right)}\right\}\times 100\;$$

The average absolute error (*AAE*) between the proposed model and the experimental data can be expressed as

(14) $$AAE\;\left(\%\right)=\frac{1}{n}\times \Sigma \left[\frac{\left|Experimental\;value-predicted\;value\right|}{Experimental\;value}\right]\times 100\;$$

where *n* is the number of test specimens.

The coefficient of determination (*R*^2^) testing the model reliability can be determined by the expression

(15) $$R^{2}=1-\frac{\Sigma {\left[Experimental\;value-predicted\;value\right]}^{2}}{\Sigma {\left[Experimental\;value-Experimental\;value_{mean}\right]}^{2}}\;$$

where the value of *R*^2^ close to 1 is considered an accurate prediction.

The slope of the best fit line (*m’*) measures the accuracy of the developed model. This slope can be readily obtained from

(16) $$m^{\prime }=\tan^{-1}\left(\frac{y_{2}-y_{1}}{x_{2}-x_{1}}\right)$$

where *x* and *y* represent the abscissa and ordinate values, respectively.

**Figure 3:**
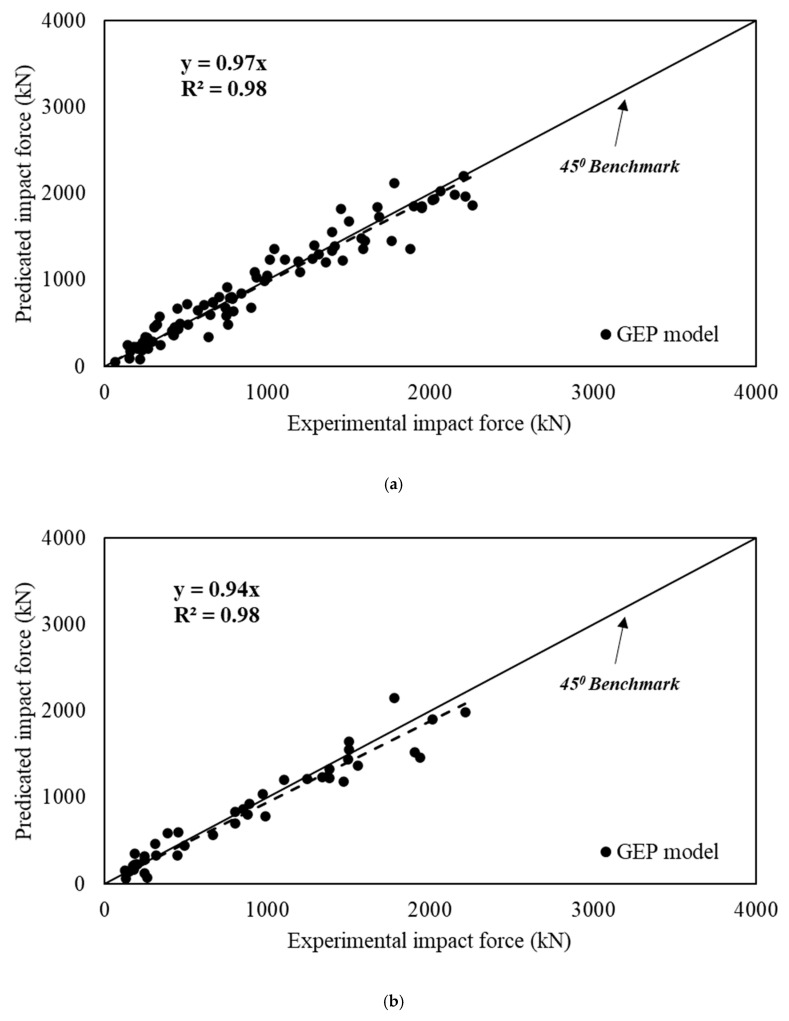

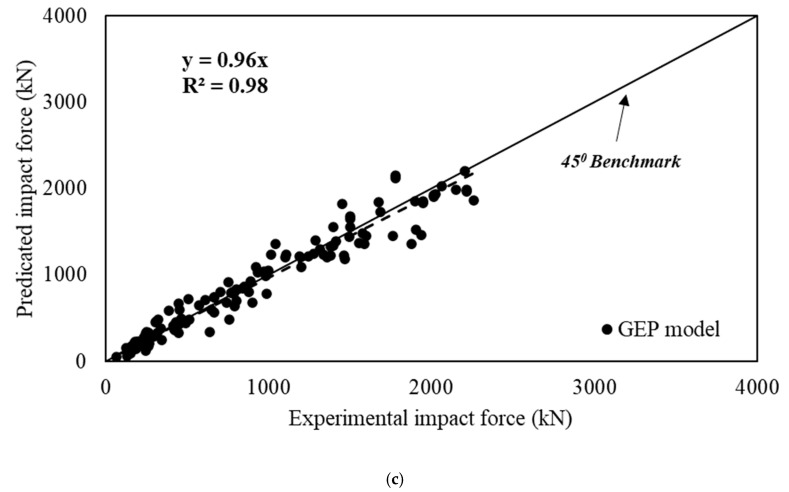
Comparison of predicted and experimental results of peak impact force. (**a**) training data; (**b**) validation data; (**c**) all data.

The statistical comparison of the proposed model with the experimental results is shown in Figure 3. The observation of the coefficient of determination (*R*^2^) suggests that its value is 0.98 for both training, validation, and overall data. Surely the close proximity of the *R*^2^ value to 1.0 suggests an accurate prediction. Furthermore, the best-fit line for the predicted peak impact force is *y* = 0.96*x* for all data, which is just under the 45° benchmark, indicating a superior correlation between experimental and predicted results.

**Figure 4:**
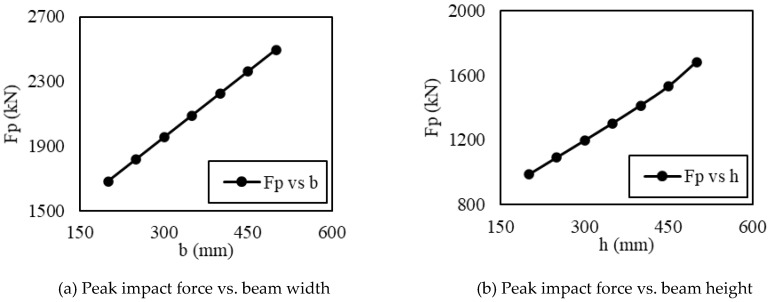

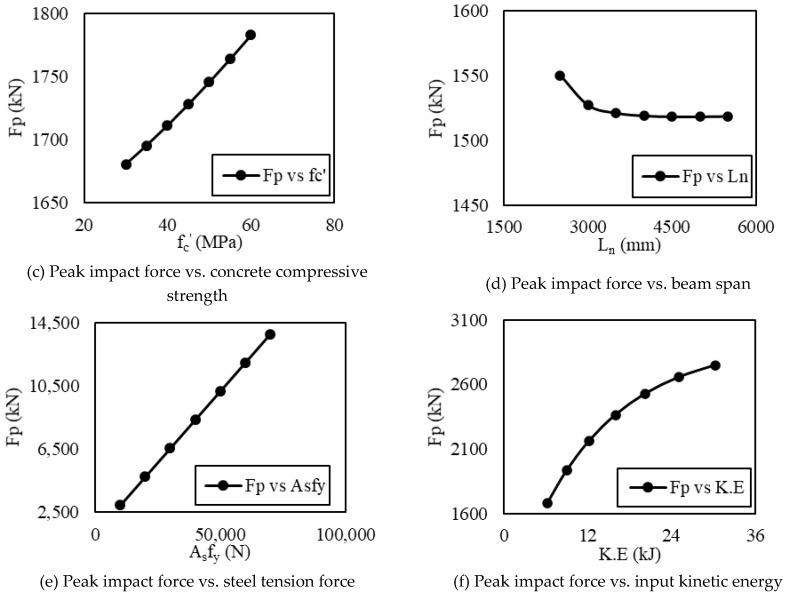
Parametric study. (**a**) Peak impact force vs. beam width; (**b**) Peak impact force vs. beam height; (**c**) Peak impact force vs. concrete compressive strength; (**d**) Peak impact force vs. beam span; (**e**) Peak impact force vs. steel tension force; (**f**) Peak impact force vs. input kinetic energy.

Understanding the influence of all the variables in the proposed model is useful for evaluating the model efficiency. Figure 4a,b show that increasing the width and depth of the beam increases the stiffness of the beam, thereby attracting more impact force. Similarly, Figure 4c shows that increasing the compressive strength of concrete increases the modulus of elasticity and the stiffness of the RC member, thus attracting more impact force. On the contrary, the peak impact force reduces as the span of the beam increases, as shown in Figure 4d. The effect of the tension force of the longitudinal reinforcement follows the pattern akin to that of Figure 4a–c,e because the ultimate bending moment capacity and the load-carrying capacity increases with the increase in the longitudinal reinforcement. The influence of input kinetic energy on the impact force is featured in Figure 4f. It can be seen that the higher magnitude of the imparted kinetic energy results in a larger impact force.

**Figure 5:**
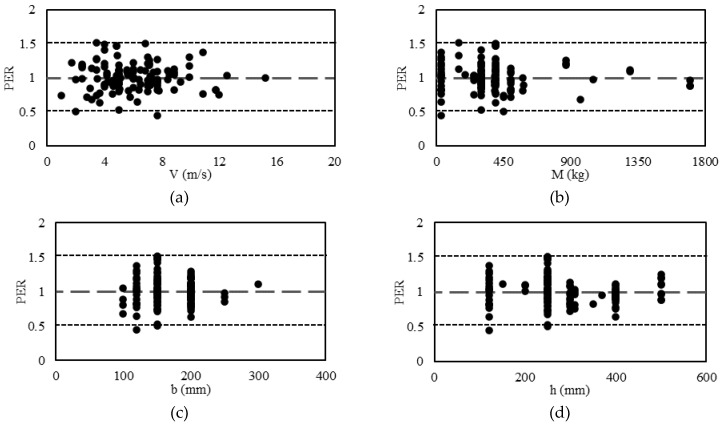

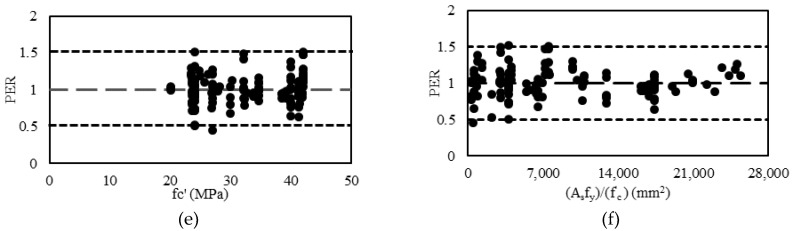
Evaluating the predictive performance of the proposed model. (**a**) Velocity of Drop, (**b**) Mass of Drop Weight, (**c**) Breath of Beam, (**d**) Depth of beam, (**e**) Concrete Compressive Strength, (**f**) Normalized Reinforcement Ration.

The predicted to the experimental ratio (PER) is an important factor in the determination of the sensitivity of various parameters in the proposed model. It is clear from Figure 5 that the PER is close to the benchmark value of 1.0 for the core variables, namely the impactor velocity *V*, the impactor mass *M*, the beam width *b*, the beam height *h*, the concrete strength $f_{c}^{\prime }$, and the ratio $\frac{A_{s}f_{y}}{f_{c}^{\prime }}$. This clearly shows the adequacy of the proposed model for the various ranges of these core variables.

## 10. Development of a Numerical Model

This section compares the proposed empirical model with a numerical model developed using finite element code ABAQUS (Version 2022, Dassault Systemes, Waltham, MA, USA). First, the experimental results of Bhatti [8] are employed to calibrate the ABAQUS model, then the impact force given by the finite element simulation is compared with the proposed GEP model. Notably, the experimental results considered herein are neither used in training nor the validation of the GEP model.

### 10.1. Experimental Program Bhatti

As per the experimental study reported by Bhatti [8], a series of 2400 mm long beams were tested under impact loading of 400 Kg. The RC beam was of 41.2 MPa compressive strength having a square cross-section of 400 × 200 mm and 50 mm cover all around. Further, the longitudinal and transverse bars were of 35 and 6 mm diameters, respectively, with the yield and ultimate strength of 395 MPa and 501 MPa, and the spacing of stirrups was 150 mm. Following Bhatti’s classification of tested specimens, the beam with stirrups spacing of 150 mm is named Type-A.

### 10.2. Response of RC Beam Predicted by ABAQUS

**Figure 6:**
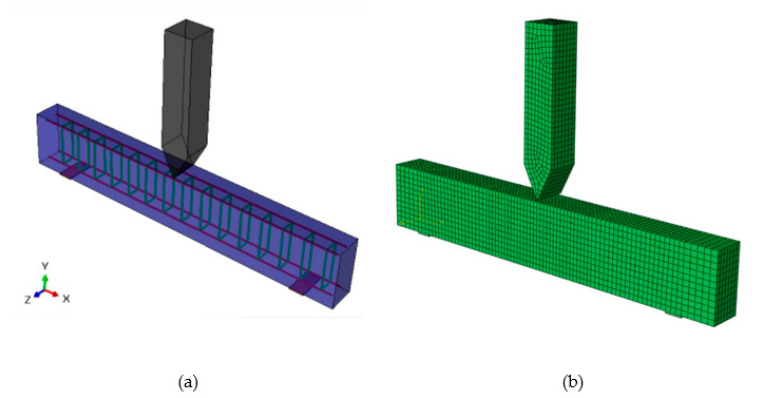
ABAQUS model of a beam subjected to central transverse impact. (**a**) FE baseline model; (**b**) FE mesh model.

A 3-D finite element model, Figure 6, was constructed to simulate the dynamic response of the RC beam. The model was developed in ABAQUS using its contact element capability. C3D8R brick elements were used for the beam, T3D2 wire elements were used to model the steel in the beam, and isoparametric elements were used to model the striker. A perfect bond between the concrete and reinforcement was ensured by adopting the embedded method. The impactor and the beam were permitted to stay in contact by employing frictionless interfacial elements. The concrete had a prescribed density of 2400 kg/m^3^, a Poisson’s ratio of 0.19, and a Young modulus of 25.7 GPa. Similarly, the reinforcement had a density of 7850 kg/m^3^, a Poisson’s ratio of 0.3, and a Young modulus of 206 GPa. The number of elements implemented for the beam was 7371 and for the impactor was 1771. The dynamic behavior was evaluated by an explicit time integration scheme with automatic control of time steps.

Mechanical properties of concrete were modeled using concrete damage plasticity, and the properties of the reinforcing bars were described by the elastic–plastic material model. Concrete damage parameters are shown in Table 3. As per the experimental study, the support conditions were simulated as simply supported. Geometrical nonlinearity was taken into account, and a convergence study was carried out to optimize the mesh size. The ABAQUS was calibrated based on the available test results of a 384 kg impactor striking the beam at midspan. ABAQUS peak impact force was determined for two test cases of impactor striking velocities, namely 6.52 m/s and 7.42 m/s.

## 11. Results and Discussions

The capability of the proposed empirical model is compared with the existing models using various statistical performance measures pointed out in Section 9. A dataset of 126 experiments was employed to examine these performance indicators.

### 11.1. Peak Impact Force on RC Beam

**Figure 7:**
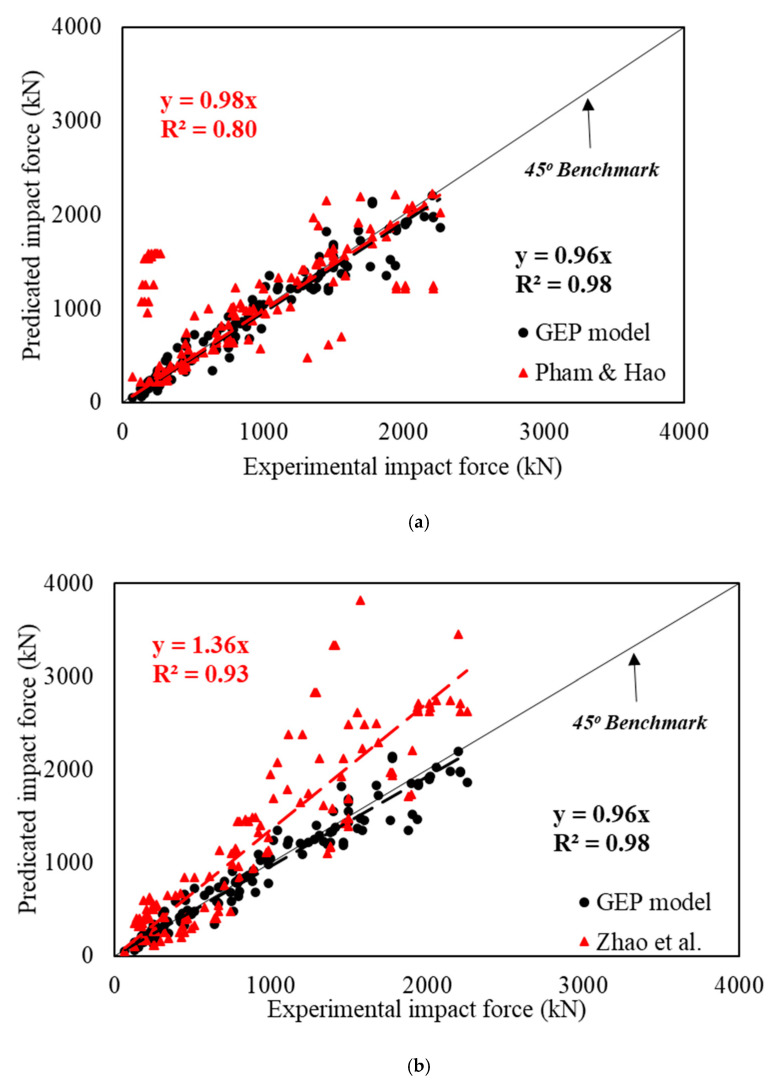
Predicted and experimental impact force proposed using the (**a**) Pham and Hao [3] and (**b**) Zhao et al. [7] models.

The prediction accuracy of the proposed model can be assessed by comparing it with the available models. Therefore, the Pham and Hao [3] and Zhao et al. [7] models are selected, and the predicted results are shown in Figure 7. The prediction of Pham and Hao [3] results in the *R*^2^ = 0.80, the average PER is 1.1, the coefficient of variation (CoV) is 116%, and the average absolute error (AAE) is 109%. Similarly, the coefficient of determination (*R*^2^) of the Zhao et al. [7] model is 0.93, with an average absolute error of 55.4%. Zhao’s model gives the average value of PER 1.43 and CoV = 41%. The proposed model has an average PER of 1.01, with a coefficient of variation (CoV) of 19% and *R*^2^ = 0.98. Having PER and *R*^2^ close to 1, and the least coefficient of variation, demonstrates the effectiveness of the GEP tool for the efficient prediction of impact force. Moreover, the average absolute error (AAE) for the proposed model is 14%, which is considerably less than the previously proposed models.

### 11.2. Numerical Verification of Empirical Model

**Figure 8:**
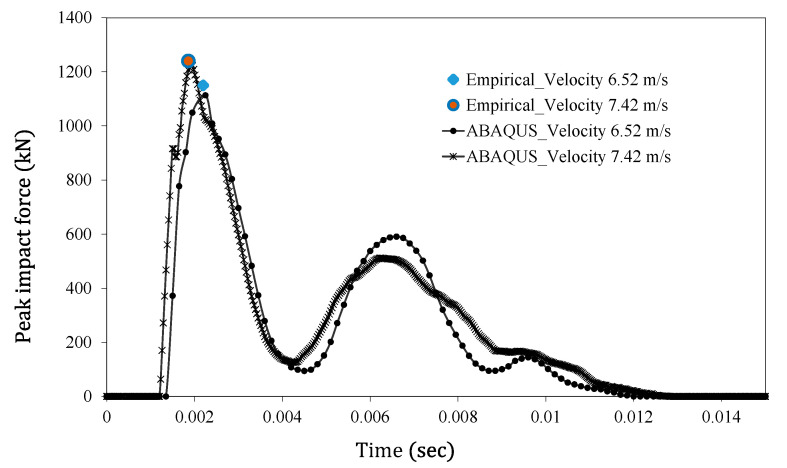
Response of RC beam model in ABAQUS: peak impact force.

The ABAQUS model was tested for two benchmark examples: the 384 kg striker impacting the beam at a velocity of 6.52 m/s and a velocity of 7.42 m/s. The most severely deformed location on the target beam is the impacted point, where the striker pierces through the beam surface. The ABAQUS results of the impact force profile are shown in Figure 8. It should be noted that the proposed GEP model predicts the peak impact force quite accurately for both cases of prescribed impactor velocity. These results agree reasonably well with the experimental results, as seen in Table 4.

### 11.3. Practical Implementation

**Figure 9:**
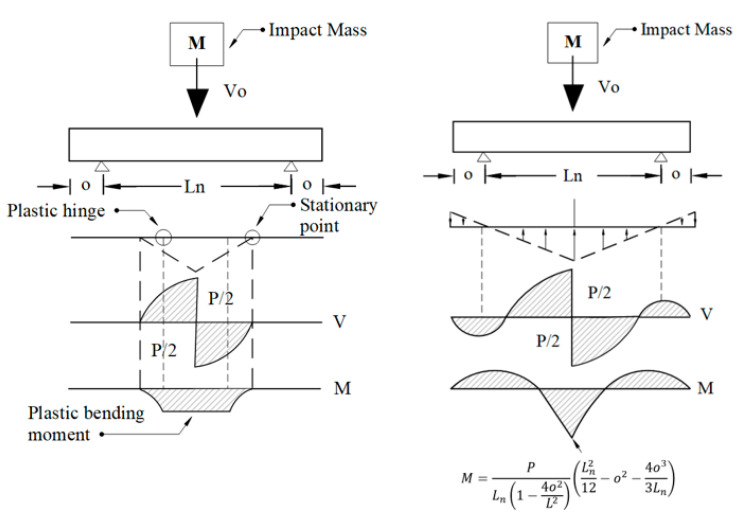
Calculation of the shear force and bending moment diagram. **M**: Impactor mass; *V*_0_: Impactor velocity; *V*: Shear force diagram; *M*: Bending moment diagram; *P*: Magnitude of shear at the point of impact; Ln: Length of beam between two supports.

The practical implementation of the peak impact force is described herein. The empirical formulation (9) can be further used for determining the dynamic shear and the bending moment diagrams of impacted RC beams. The full procedure shown in Figure 9 for generating this response is given by Pham et al. [18]. According to this procedure, these diagrams can be reasonably predicted provided the maximum impact force and the location of the plastic hinges are known. Having the maximum impact force determined from (5) and the hinge locations estimated from the model proposed by Pham et al. [44], the shear force and bending moment diagrams can be easily generated. These diagrams can be readily implemented to design beams under extreme dynamic loading.

## 12. Conclusions

The peak impact force is one of the most important response parameters governing the overall dynamic response of the impacted beam. An evolutionary genetic model is developed to predict this important force that is transferred to the RC beam under impact loading. The key contribution of this model is the implementation of key influencing factors including concrete compressive strength, the shear span to depth ratio, and the tensile reinforcement quantity and strength, which has considerably improved the accuracy as compared to the other available models. Based on the available literature, 126 experiments were compiled, of which 42 experiments were employed for validation purposes. The proposed machine learning model is benchmarked against two existing models, one analytical and the other regression-based. The following conclusions are drawn:

- An increase in the influencing factors, such as the concrete compressive strength, the steel bar tensile strength, the longitudinal reinforcement ratio, the geometric dimension of beam cross-section, and the input kinetic energy results in attracting more impact force. Contrarily, an increase in the beam span results in attracting less impact force;
- The proposed empirical equation can accurately determine the impact force by incorporating all the aforementioned influencing factors. In addition, the influencing parameters in the model are consistently related to the experimental data;
- The predictive ability of the proposed model is indicated by the coefficient of determination, performance factor, and the average absolute error (98%, 1.01, and 15.2%, respectively);
- The proposed model can approximate the transmitted impact force to the RC beam more closely than an available analytical model and an artificial neural network-based model. A comparison is made with two different models to evidence the predictive ability of the developed model;
- The proposed model is also validated by comparing the results with the FE simulation, using an ABAQUS/Explicit solver, which was developed on existing experimental results. The model gives good agreement with the numerical results showing less than 4% error;
- Overall, the proposed regression model offers an excellent predictive tool for determining the peak impact force transmitted to RC beams subjected to impact. Surely, the predictability of the current model has been improved by incorporating the important influencing parameters, such as the properties of shear and longitudinal reinforcement. This impact force can be employed for determining the bending and shear design of these beams, which is useful design information in terms of sizing and detailing of RC beams under impact loading;
- In future work, experiments will be performed to further validate the empirical formulation with varying impactor mass, striking velocity, and steel reinforcement in an RC beam. Additionally, the ABAQUS formulation will be improved by adopting actual stress–strain curves to accurately simulate the tested beams.

## Figures and Tables

**Table 1:** Distribution of key influence parameters.

Parameters	Ranges
Velocity (*V*)	1–16 (m/s)
Mass (*M*)	33–1700 (kg)
$\mathrm{Compressive}\;\mathrm{strength}\;(f_{c}^{\prime }$)	20–42 (MPa)
Net span length (*L_n_*_)_	1000–5000 (mm)
Beam width (*b*)	100–300 (mm)
Beam height (*h*)	120–500 (mm)
Tensile reinforcement ratio	0.29–3.1 (%)
Shear reinforcement ratio	0–1.4 (%)

**Table 2:** Model Construction Parameters.

Function Set	ln, +, −, /, x, sqrt, *x*^2,3,4,5^
Chromosomes	110
Head size	11
Linking function	Addition
Number of genes	3
Mutation rate	0.0014
Inversion rate	0.1
One-point recombination rate	0.0027
Two-point recombination rate	0.0027
Gene recombination rate	0.0027
Gene transposition rate	0.0027
Constants per gene	10
Lower/upper bound of constants	−20/20

**Table 3:** Parameters of concrete damage plasticity model.

Parameters	Values
$\mathrm{Tensile}\;\mathrm{strength}\;\mathrm{of}\;\mathrm{concrete}\;0.1\times f_{c}^{\prime }$ (MPa)	4.12
Dilation angle (degrees)	35
Eccentricity	0.10
Bi-axial to uni-axial strength ratio	1.16
Second stress invariant ratio	0.667
Viscosity parameter	0.01

**Table 4:** Comparison of GEP result with experimental and ABAQUS results.

	Experimental Results	ABAQUS Results	GEP Results
Peak Impact force on the beam (kN) [Striker velocity 6.52 m/s]	1110	1113	1150
Peak Impact force on the beam (kN) [Striker velocity 7.42 m/s]	1290	1229	1240

## Data Availability

Data will be made available on request.

## Appendix A

**Table A1:** Beam geometry and reinforcement details of RC beams.

ID	Geometric Size					Concrete	Longitudinal Reinforcement				Shear Reinforcement	
	b (mm)	h (mm)	L (mm)	L_n_ (mm)	a (mm)	F^’^_c_ (Mpa)	A_s_ (mm^2^)	F_y_ (Mpa)	A^’^_s_ (mm)	F^’^_y_ (Mpa)	F_vy_ (Mpa)	Ratio %
A37	200	400	2400	2000	50	41.2	1924	373	1924	373	373	0.21
A46	200	400	2400	2000	50	41.2	1924	373	1924	373	373	0.21
A56	200	400	2400	2000	50	41.2	1924	373	1924	373	373	0.21
A65	200	400	2400	2000	50	41.2	1924	373	1924	373	373	0.21
A74	200	400	2400	2000	50	41.2	1924	373	1924	373	373	0.21
A84	200	400	2400	2000	50	41.2	1924	373	1924	373	373	0.21
B37	200	400	2400	2000	50	41.2	1924	373	1924	373	373	0.42
B46	200	400	2400	2000	50	41.2	1924	373	1924	373	373	0.42
B65	200	400	2400	2000	50	41.2	1924	373	1924	373	373	0.42
B74	200	400	2400	2000	50	41.2	1924	373	1924	373	373	0.42
B84	200	400	2400	2000	50	41.2	1924	373	1924	373	373	0.42
B93	200	400	2400	2000	50	41.2	1924	373	1924	373	373	0.42
S1616-1	150	250	1700	1400	40	42.0	402	426	402	426	295	1.40
S1616-2	150	250	1700	1400	40	42.0	402	426	402	426	295	1.40
S1616-3	150	250	1700	1400	40	42.0	402	426	402	426	295	1.40
S1616-4	150	250	1700	1400	40	42.0	402	426	402	426	295	1.40
S1322-1	150	250	1700	1400	40	42.0	760	397	265	397	295	1.40
S1322-2	150	250	1700	1400	40	42.0	760	397	265	397	295	1.40
S1322-3	150	250	1700	1400	40	42.0	760	397	265	397	295	1.40
S1322-4	150	250	1700	1400	40	42.0	760	397	265	397	295	1.40
S2222-1	150	250	1700	1400	40	42.0	760	418	760	418	295	1.40
S2222-2	150	250	1700	1400	40	42.0	760	418	760	418	295	1.40
S2222-3	150	250	1700	1400	40	42.0	760	418	760	418	295	1.40
S2222-4	150	250	1700	1400	40	42.0	760	418	760	418	295	1.40
A1	150	250	1400	1000	40	24.0	265	345	265	345	295	0.75
A2-1	150	250	2400	2000	40	24.0	265	345	265	345	295	0.75
A2-2	150	250	2400	2000	40	24.0	265	345	265	345	295	0.75
A2-3	150	250	2400	2000	40	24.0	265	345	265	345	295	0.75
A2-4	150	250	2400	2000	40	24.0	265	345	265	345	295	0.75
A2-5	150	250	2400	2000	40	24.0	265	345	265	345	295	0.75
A2-6	150	250	2400	2000	40	24.0	265	345	265	345	295	0.75
A2-7	150	250	2400	2000	40	24.0	265	345	265	345	295	0.75
A2-8	150	250	2400	2000	40	24.0	265	345	265	345	295	0.75
A2-9	150	250	2400	2000	40	24.0	265	345	265	345	295	0.75
A2-10	150	250	2400	2000	40	24.0	265	345	265	345	295	0.75
A2-11	150	250	2400	2000	40	24.0	265	345	265	345	295	0.75
A2-12	150	250	2400	2000	40	24.0	265	345	265	345	295	0.75
A2-13	150	250	2400	2000	40	24.0	265	345	265	345	295	0.75
A2-14	150	250	2400	2000	40	24.0	265	345	265	345	295	0.75
A4	150	250	4400	4000	40	24.0	265	345	265	345	295	0.75
B	300	150	2400	2000	40	24.0	530	345	530	345	295	0.75
C	150	250	2400	2000	40	24.0	402	345	402	345	295	0.75
D	150	250	2400	2000	40	24.0	157	345	157	345	295	0.75
E	150	400	2400	2000	40	24.0	265	345	265	345	295	0.75
F	150	400	2400	2000	40	24.0	157	345	157	345	295	0.75
G1-1	200	300	3400	3000	40	33.7	567	379	567	379	295	0.07
G1-1S	200	300	3400	3000	40	33.7	567	379	567	379	295	0.07
G2-1	150	250	2400	2000	40	32.2	265	373	265	373	295	0.13
G2-2	150	250	2400	2000	40	32.2	265	373	265	373	295	0.13
G2-3	150	250	2400	2000	40	32.2	265	373	265	373	295	0.13
G2L-1	150	250	2400	2000	40	32.2	265	373	265	373	295	0.13
G2L-2	150	250	2400	2000	40	32.2	265	373	265	373	295	0.13
G2L-3	150	250	2400	2000	40	32.2	265	373	265	373	295	0.13
G3-1	150	250	2400	2000	40	34.6	265	393	265	393	295	0.13
G3-2	150	250	2400	2000	40	34.6	265	393	265	393	295	0.13
G3-3	150	250	2400	2000	40	34.6	265	393	265	393	295	0.13
G4-1	150	250	2400	2000	40	32.3	265	373	265	373	295	0.13
G4-2	150	250	2400	2000	40	32.3	265	373	265	373	295	0.13
G5-1	200	300	3400	3000	40	39.2	567	379	567	379	295	0.07
G5-2	200	300	3400	3000	40	39.2	567	379	567	379	295	0.07
G6-1	250	250	2400	2000	40	34.7	567	392	567	392	295	0.13
G7-1	250	250	3400	3000	40	34.7	567	392	567	392	295	0.09
G7-2	250	250	3400	3000	40	34.7	567	392	567	392	295	0.09
G8-1	200	200	2400	2000	40	34.7	982	383	982	383	295	0.16
G9-1	200	200	3400	3000	40	34.7	982	383	982	383	295	0.12
G9-2	200	200	3400	3000	40	34.7	982	383	982	383	295	0.12
G10-1	200	250	3400	3000	40	23.5	567	404	567	404	295	0.09
G10-2	200	250	3400	3000	40	23.5	567	404	567	404	295	0.09
G10-3	200	250	3400	3000	40	23.5	567	404	567	404	295	0.09
G10-4	200	250	3400	3000	40	23.5	567	404	567	404	295	0.09
G11-1	200	300	3100	2700	50	23.6	760	401	760	401	295	0.08
G11-2	200	300	3100	2700	50	23.6	760	401	760	401	295	0.08
G11-3	200	300	3100	2700	50	23.6	760	401	760	401	295	0.08
G11-4	200	300	3100	2700	50	23.6	760	401	760	401	295	0.08
G11-5	200	300	3100	2700	50	23.6	760	401	760	401	295	0.08
G11-6	200	300	3100	2700	50	23.6	760	401	760	401	295	0.08
G12-1	200	300	3100	2700	50	23.6	1473	407	1473	407	295	0.08
G13-1	200	400	3100	2700	50	23.6	982	406	982	406	295	0.06
G14-1	200	350	3100	2700	50	23.6	982	406	982	406	295	0.07
G15-1	200	400	3100	2700	50	23.6	982	406	982	406	295	0.06
G16-1	200	370	3100	2700	50	23.6	982	406	982	406	295	0.06
B-1700-4.60	200	500	6000	5000	40	27.0	1257	495	402	495	344	0.09
B-1052-6.40	200	500	6000	5000	40	27.9	1257	495	402	495	344	0.09
B-868-7.14	200	500	6000	5000	40	24.8	1257	495	402	495	344	0.09
C-1700-4.60	200	500	4000	3000	40	32.1	1257	495	402	495	344	0.09
C-1300-5.56	200	500	4000	3000	40	30.3	1257	495	402	495	344	0.09
C-868-7.14	200	500	4000	3000	40	26.3	1257	495	402	495	344	0.09
D-1700-4.60	200	500	4000	3000	40	32.7	1257	495	402	495	344	0.19
D-1300-5.56	200	500	4000	3000	40	25.6	1257	495	402	495	344	0.19
D-868-7.14	200	500	4000	3000	40	25.0	1257	495	402	495	344	0.19
BD1	150	310	2700	1860	33	28.1	603	477	603	477	550	0.22
BD2	150	310	2700	1860	33	26.9	603	477	603	477	550	0.22
BD3	150	310	2700	1860	33	26.9	603	477	603	477	550	0.22
BD4	150	310	2700	1860	33	41.4	603	477	603	477	550	0.22
BD5	150	310	2700	1860	33	41.4	603	477	603	477	550	0.22
A-1	100	250	3000	2500	28	30.0	402	490	402	490	340	0.38
A-2	100	250	3000	2500	28	30.0	402	490	402	490	340	0.38
A-3	100	250	3000	2500	28	30.0	402	490	402	490	340	0.38
A-4	100	250	3000	2500	28	30.0	402	490	402	490	340	0.38
B-0-00	200	400	2800	2400	42.5	41.9	1473	460	402	460	0	0.00
B-0-25	200	400	2800	2400	42.5	41.9	1473	460	402	460	314	0.25
B-0-50	200	400	2800	2400	42.5	41.9	1473	460	402	460	314	0.50
BD1	150	310	3000	1860	33	45.8	603	394	0	394	0	0.00
BD2	150	310	3000	1860	33	45.8	603	394	0	394	0	0.00
BD3	150	310	3000	1860	33	45.8	603	394	0	394	0	0.00
BD4	150	310	3000	1860	33	45.8	603	394	0	394	0	0.00
BD5	150	310	3000	1860	33	45.8	603	394	0	394	0	0.00
B1	200	400	2800	2400	42	20.1	982	429	402	429	314	0.50
B2	200	400	2800	2400	42	20.1	982	429	402	429	314	0.50
B3	200	400	2800	2400	42	20.1	982	429	402	429	314	0.50
B4	200	400	2800	2400	42	38.5	1473	429	402	429	0	0.00
B5	200	400	2800	2400	42	38.5	1473	429	402	429	314	0.50
B6	200	400	2800	2400	42	38.5	1473	429	402	429	314	0.25
10-C27-4	120	120	1800	1200		27	157	235	157	235	235	0.5
6-C40-5	120	120	1800	1200		40	56.52	235	56.52	235	235	0.5
6-C40-6	120	120	1800	1200		40	56.52	235	56.52	235	235	0.5
6-C40-7	120	120	1800	1200		40	56.52	235	56.52	235	235	0.5
6-C40-8	120	120	1800	1200		40	56.52	235	56.52	235	235	0.5
8-C40-2	120	120	1800	1200		40	101	235	101	235	235	0.5
8-C40-3	120	120	1800	1200		40	101	235	101	235	235	0.5
8-C40-4	120	120	1800	1200		40	101	235	101	235	235	0.5
8-C40-5	120	120	1800	1200		40	101	235	101	235	235	0.5
10-C40-5	120	120	1800	1200		40	157	235	157	235	235	0.5
10-C40-6	120	120	1800	1200		40	157	235	157	235	235	0.5
10-C40-7	120	120	1800	1200		40	157	235	157	235	235	0.5

**Table A2:** Impact response characteristics.

ID	Hammer		Test Results			Reference
	V (m/s)	M (kg)	F_P_ (kN)	S_max_ (mm)	Failure Model	
A37	3.67	400	750	8.1	Flexure	[8]
A46	4.58	400	880	10.1	Flexure	
A56	5.61	400	1000	13.1	Flexure-shear	
A65	6.52	400	1110	16.0	Shear	
A74	7.42	400	1290	22.0	Shear	
A84	8.4	400	1400	24.4	Shear	
B37	3.67	400	760	7.5	Flexure	
B46	4.58	400	900	11.3	Flexure-shear	
B65	6.52	400	1200	16.0	Shear	
B74	7.42	400	1280	19.8	Flexure-shear	
B84	8.4	400	1416	24.0	Flexure-shear	
B93	9.3	400	1575	28.0	Flexure-shear	
S1616-1	1.73	400	125	5.9	None	[4]
S1616-2	2.45	400	180	11.0	None	
S1616-3	3.46	400	248	20.1	Flexure	
S1616-4	4.85	400	317	36.2	Flexure	
S1322-1	2.45	400	190	7.8	None	
S1322-2	3.46	400	260	11.4	Flexure	
S1322-3	4.85	400	305	23.5	Flexure	
S1322-4	6.86	400	340	27.0	Flexure	
S2222-1	2.45	400	200	7.0	None	
S2222-2	3.46	400	260	11.0	Flexure	
S2222-3	4.85	400	313	21.4	Flexure	
S2222-4	6.86	400	388	31.8	Flexure	
A1	5	300	434	24.1	n/p	[23]
A2-1	3.46	150	321	13.6	Flexure	
A2-2	2.45	300	293	25.4	Flexure	
A2-3	2	450	245	37.0	Flexure	
A2-4	4.9	150	453	16.3	Flexure	
A2-5	3.5	300	417	31.6	Flexure	
A2-6	2.8	450	346	43.7	Flexure	
A2-7	6	150	573	17.9	Flexure	
A2-8	4.24	300	513	33.3	Flexure	
A2-9	3.46	450	445	48.4	Flexure	
A2-10	1	300	65	4.5	Flexure	
A2-11	2	300	253	12.6	Flexure	
A2-12	3	300	426	26.9	Flexure	
A2-13	4	300	489	41.4	Flexure	
A2-14	5	300	466	58.3	Flexure	
A4	5	300	452	114.9	n/p	
B	5	300	667	77.0	n/p	
C	5	300	650	42.4	n/p	
D	5	300	639	94.0	n/p	
E	5	300	742	29.1	n/p	
F	5	300	664	43.9	n/p	
G1-1	7	300	1496	64.3	n/p	[2]
G1-1S	7	300	1601	58.0	n/p	
G2-1	4	300	510	28.3	n/p	
G2-2	5	300	779	44.0	n/p	
G2-3	6	300	854	57.0	n/p	
G2L-1	4	400	447	44.2	Flexure	
G2L-2	5	400	668	66.8	Flexure	
G2L-3	6	400	787	89.7	Flexure	
G3-1	4	300	609	36.7	n/p	
G3-2	5	300	770	52.0	n/p	
G3-3	6	300	839	70.6	n/p	
G4-1	4	300	800	39.7	n/p	
G4-2	5	300	986	56.1	n/p	
G5-1	6	400	1313	63.5	Flexure	
G5-2	7	400	1557	83.4	Flexure	
G6-1	5	300	1336	26.4	n/p	
G7-1	5	300	1243	45.8	n/p	
G7-2	6	300	1588	60.9	n/p	
G8-1	6	300	1192	36.5	n/p	
G9-1	5	300	931	43.2	n/p	
G9-2	6	300	1103	57.9	n/p	
G10-1	4	300	751	33.7	None	
G10-2	5	300	922	49.5	Flexure	
G10-3	6	300	1017	67.8	Flexure	
G10-4	7	300	1042	83.9	Flexure	
G11-1	3.13	500	703	20.5	n/p	
G11-2	4.2	500	971	33.2	n/p	
G11-3	5.05	500	1462	43.1	n/p	
G11-4	5.78	500	1878	55.5	n/p	
G11-5	6.42	500	1765	67.2	n/p	
G11-6	7	500	1907	83.4	n/p	
G12-1	7.67	500	1675	85.4	n/p	
G13-1	7.67	500	2150	60.6	n/p	
G14-1	7.67	500	2258	63.7	n/p	
G15-1	7.67	500	2063	40.5	n/p	
G16-1	7.67	500	2023	52.9	n/p	
B-1700-4.60	4.6	1700	1360	83.7	Flexure	[24]
B-1052-6.40	6.4	1052	1900	79.3	Flexure-shear	
B-868-7.14	7.14	868	1450	209.0	Shear	
C-1700-4.60	4.6	1700	1380	118.0	Shear	
C-1300-5.56	5.56	1300	1500	67.4	Shear	
C-868-7.14	7.14	868	1780	67.9	Shear	
D-1700-4.60	4.6	1700	1380	41.5	Flexure	
D-1300-5.56	5.56	1300	1500	48.0	Flexure	
D-868-7.14	7.14	868	1780	47.5	Flexure-shear	
BD1	4.15	253	891	12.0	Flexure	[40]
BD2	7.11	253	1396	24.0	Flexure	
BD3	11.96	253	1940	75.0	Shear	
BD4	7.81	578	1466	80.0	Flexure	
BD5	5.1	578	983	38.0	Flexure-shea	
A-1	3.12	968	260	81.0	Flexure	[41]
A-2	4	587	420	74.0	Flexure	
A-3	5.7	392	790	83.6	Flexure	
A-4	8.5	197	800	89.5	Flexure	
B-0-00	7.2	393	2014	142.6	Shear	[39]
B-0-25	7.2	393	2214	31.4	Flexure-shear	
B-0-50	7.2	393	1948	20.3	Flexure	
BD1	4.43	333	912	n/p	Shear	[49]
BD2	8.85	333	1708	n/p	Shear	
BD3	5.71	200	1184	n/p	Shear	
BD4	4.43	200	768	n/p	Flexure	
BD5	5.71	200	1360	n/p	Flexure-shea	
B1	5.9	253	1500	12.4	Flexure-shear	[42]
B2	15.2	253	2203	79.8	Flexure	
B3	7.2	393	1689	44.8	Flexure	
B4	7.2	393	2014	142.6	Shear	
B5	7.2	393	1948	20.3	Flexure	
B6	7.2	393	2214	31.4	Flexure-shear	
10-C27-4	8.85	33.6	141.97	28.66	n/p	[38]
6-C40-5	9.9	33.6	202.2	62.65	n/p	
6-C40-6	10.84	33.6	267.7	87.74	n/p	
6-C40-7	11.71	33.6	230.71	119.07	n/p	
6-C40-8	12.52	33.6	224.75	119.11	n/p	
8-C40-2	6.26	33.6	151.73	22.94	n/p	
8-C40-3	7.67	33.6	182.59	35.65	n/p	
8-C40-4	8.85	33.6	203.26	35.14	n/p	
8-C40-5	9.9	33.6	234.5	38.82	n/p	
10-C40-5	9.9	33.6	245.8	33.7	n/p	
10-C40-6	10.84	33.6	248.56	45.22	n/p	
10-C40-7	11.71	33.6	183.3	62.5	n/p	
